# Reaction mechanism, cure behavior and properties of a multifunctional epoxy resin, TGDDM, with latent curing agent dicyandiamide†

**DOI:** 10.1039/c7ra13233f

**Published:** 2018-02-21

**Authors:** Feng Wu, Xingping Zhou, Xinhai Yu

**Affiliations:** College of Chemistry, Chemical Engineering and Biotechnology, Donghua University Shanghai 201620 P. R. China wufeng@mail.dhu.edu.cn yuxinhai@dhu.edu.cn xpzhou@dhu.edu.cn +86 021 67792601

## Abstract

A novel resin system was prepared using the glycidyl amide type multifunctional epoxy resin *N*,*N*,*N*′,*N*′-tetraglycidyl-4,4′-diaminodiphenylmethane (TGDDM) and latent curing agent dicyandiamide (DICY). The curing reaction mechanism of the TGDDM/DICY system was studied by Fourier transform infrared (FTIR) spectrometry and the non-isothermal cure behaviors of the mixture were investigated with differential scanning calorimetry (DSC) measurements. The FTIR results demonstrated that there were two main reactions occurring in the curing process of the TGDDM/DICY system. The DSC thermogram of the blend exhibited two different cure regimes in the temperature range of 140–358 °C, and the system experienced two autocatalytic curing processes with *α* = 0.45 as the boundary; the corresponding average activation energies calculated by the Kissinger method were 69.7 and 88.7 kJ mol^−1^, respectively. In addition, the correlation between activation energy *E*_a_ and fractional conversion *α* was determined by applying model-free isoconversional analysis with Flynn–Wall–Ozawa (FWO) and Starink methods. Results showed that both methods revealed similar trends and possessed approximately the same values at each fractional conversion. Activation energy varied greatly with fractional conversion and the possible causes behind the variations were analyzed in detail. The cured TGDDM/DICY exhibited outstanding mechanical and adhesive properties with tensile and shear strengths of 27.1 MPa at 25 °C and12.6 MPa at 200 °C, good dielectric properties with a low dielectric constant of 3.26 at 1000 kHz and a low water absorption of 0.41%.

## Abstract 2

The curing mechanism of the TGDDM/DICY system consisted of two main reactions and it experienced two autocatalytic curing processes.

## Graphical abstract

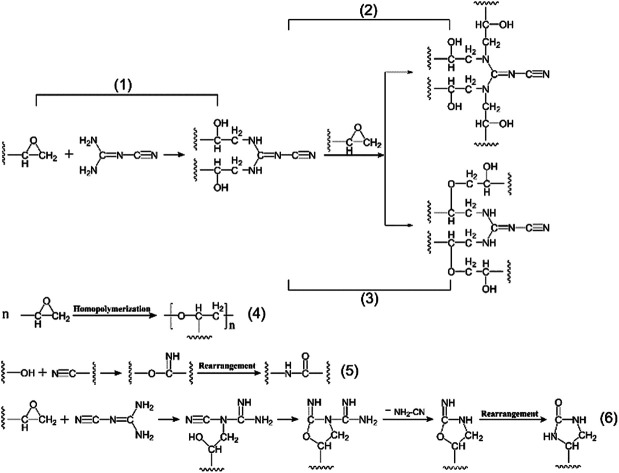

## 1. Introduction

As one of the most important classes of high performance thermosetting polymers, epoxy resins have been widely applied as composite matrices, electronic-packaging mold compounds, insulating and embedding materials, protective coatings, adhesives, sealants, and so on. This is due to their attractive properties such as strong adhesion to various substrates, high chemical and corrosion resistance, good mechanical and electrical properties, low cure shrinkage and formulation diversity.^1–5^ With the rapid development of diverse high-tech fields, there is a growing demand for advanced epoxy resins, which possess outstanding overall performance, especially adequate thermal and mechanical properties to meet the increasingly complex requirements of high performance structural products. Currently, a number of nonlinear multifunctional epoxy resins play important roles in advanced epoxy resins and attract considerable interest among academia.^6–8^ The multifunctional glycidyl amide type epoxy resin *N*,*N*,*N*′,*N*′-tetraglycidyl-4,4′-diaminodiphenylmethane (TGDDM) is a successful example. Thanks to four nonlinear functional groups and high crosslink density of its cured products, TGDDM owns lower viscosity, better flowability and processability, outstanding thermo-mechanical performance when compares with the conventional linear bisphenol-A type epoxy resins, and has been extensively used in application areas ranging from aircraft and aerospace industries to electronic/electrical and other related high-tech fields.^9,10^

In practical applications, to achieve above superior all-round properties, a curing process is desperately needed to convert the original epoxy resins from monomers and/or oligomers into a permanently highly crosslinked three-dimensional network macromolecule in the presence of suitable curing agents under optimal curing and processing conditions.^11^ Furthermore, curing agents play a crucial part in determining fundamental curing reaction mechanisms, curing conditions, processability, pot life, cured network structures, end-use properties and ultimate practical application fields of the final epoxy materials.^12^ Frequently used epoxy curing agents include amines, modified amines, acid anhydrides, phenolic resins, *etc.* Because of the violent exothermic reaction and short storage period, most of the traditional curing agents and the crude epoxide have to be stored separately and mix immediately before use, which not only pollute the environment but also strongly affect the processability, production efficiency and application. Recently, numerous latent curing reagents that can be packaged together with epoxides at ambient temperature have been developed.^13,14^ Dicyandiamide (DICY) is a solid powder with a limited solubility in epoxides at room temperature, which can endow its epoxy prepolymers with excellent processability and room-temperature stability and the corresponding cured products with wonderful mechanical and electrical properties. For these reasons, DICY is resoundingly used as a thermally latent curing agent for epoxy resins in laminates, prepregs, coatings and adhesives.^15–17^

Generally, the final properties of the cured epoxy materials are determined mainly by the network macromolecular structures and morphology, which depend on the type of cure reaction and the stage that it takes place during the curing process.^18,19^ Therefore, it is imperative to study the reaction mechanism and curing behaviors of epoxy systems. To date, many research efforts have been devoted to the research of epoxy/DICY systems in or without the presence of accelerator, toughener and other additives.^20,21^ However, there are still disputations in respect of the cure mechanism, a clear understanding of the curing reaction mechanism has still not been achieved. It is actually not surprising when one ascribes to the multiplicity and complexity of the epoxy/DICY formulations. According to the reports,^22–28^ the following several reactions were proposed in the curing process of epoxy/DICY system: the ring-opening addition reaction of epoxy ring and primary amine for producing chain growth and secondary amine for building chain branches, etherification of epoxy ring with pendant hydroxyl group, homopolymerization of unreacted epoxy group at higher temperature in the absence of active N–H functionality, addition reaction between nitrile group in DICY and resultant hydroxyl group or excess epoxy group to generate imine and further rearrangement reaction to yield amide. In principle, the above-mentioned reactions may occur, either simultaneously or at different stages of the curing process, depending on the relative reactivity of the components and on the process temperature. Such as the undoped epoxy/diamine system, at lower temperature the ring-opening reaction of epoxy ring and primary amine is the exclusive one occurring in the presence of primary amine. Only when the concentration of primary amine group becomes negligible, the reactions between secondary amine or pendant hydroxyl group and epoxy ring start to happen, whereas the three reactions may take place almost simultaneously with increasing process temperature.^29^ Furthermore, the unreacted epoxy group trapped in glassy polymeric network may self-polymerize to form the polyether under the catalysis of resultant tertiary amine group at higher temperature.^18,30,31^

In fact, a great majority of epoxy resin references focuses on various bisphenol-A diglycidyl ether type epoxy resin (DGEBPA),^32^ while there are very limited investigations concerning high performance advanced epoxy resins, particularly, no systematic studies on the reaction mechanism and cure behaviors of multifunctional tetraglycidyl amide type epoxy resin TGDDM cured with latent curing agent DICY. Therefore, the present work aims to make up for the vacancy. The curing reaction mechanism and cure behaviors of TGDDM/DICY system were evaluated by FTIR and non-isothermal DSC, respectively. Finally, property investigations of the TGDDM/DICY were exhibited, including viscosity, tensile and shear property, dielectric property and water absorption.

## 2. Kinetics theory

It is known that the crosslinking of linear macromolecules with complicated mechanism occurs in the curing process of epoxy resin and the cure reactions of epoxy systems are exothermic. In this instance, DSC instrument that is able to record reaction heat throughout the dynamic or isothermal cure experiments is very suitable for analyzing reaction kinetics of epoxy polymerization. In the DSC kinetic studies of thermosetting resins, the basic assumptions are that the heat evolution recorded by DSC is proportional to the extent of consumption of reactive groups (or the fractional conversion), and that the reaction rate is proportional to the measured heat flow. The heat flow (*Q*), as a function of temperature and time, is directly recorded by DSC and then be further processed to obtain fractional conversion (*α*) and reaction rate (d*α*/d*t*).^33^ Accordingly, *α*(*t*) and d*α*/d*t* can be described as follows:1
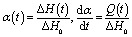
where *t* is the reaction time, Δ*H*_0_ is the total reaction heat in the system. Δ*H*(*t*) is the amount of heat released until the time *t* and can be directly determined by the integral of heat flow *Q*(*t*):2
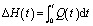


For dynamic DSC cure experiments with identical heating rates, the above integral can alternatively expressed as:3
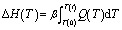
where *T* is the absolute temperature, *β* = d*T*/d*t* is the heating rate used in the DSC test.

The rate equation in kinetics analysis can also be described by eqn (4):4
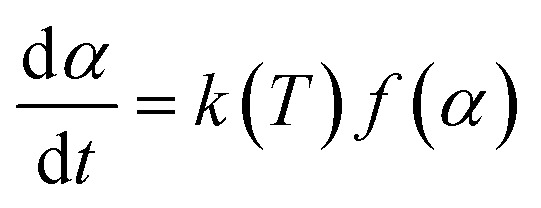
where *f*(*α*) is a function of fractional conversion associated with the reaction mechanism. *k*(*T*) is a temperature-dependent reaction rate constant that follows the Arrhenius equation as shown in eqn (5).5
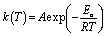
where *A* is the pre-exponential factor or frequency factor, *E*_a_ is the curing activation energy and *R* is the universal gas constant.

So far, a considerable number of calculation methods have been used to analyze the activation energy of curing reaction of epoxy resin. The Kissinger equation, as one of the most frequently cited methods, has been employed to evaluate the activation energy of non-isothermal reactions in academia. It assumes that the maximum reaction rate occurs at the peak temperature and determines the activation energy simply without beforehand precise knowledge of the reaction mechanism.^34^ The Kissinger equation is expressed as eqn (6). According to this method, *E*_a_ is obtained from the maximum reaction rate and calculated from the slope of the straight line which acquired by plotting of ln(*β*/*T*_p_^2^) against 1/*T*_p_.6
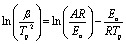


Inaccurate value of *E*_a_ can be resulted from a rough temperature integral approximation. A modified integral method proposed by M. J. Starink based on Kissinger–Akahira–Sunose method,^35^ which is of more accurate, is used to estimate the activation energies (*E*_a_) for different conversion degrees (*α*). Starink equation is expressed as follow:7
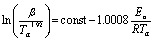


It determines the activation energy by the plot of ln(*β*/*T*_*α*_^1.92^) against 1/*T*_*α*_ for a constant value *α*. Where *T*_p_ is the absolute exothermic peak temperature, *T*_*α*_ is the absolute temperature at a fixed value of the variable *α*.

Independently, the Flynn–Wall–Ozawa method is a model-free isoconversional method for non-isothermal data, which assumes that both activation energy and pre-exponential factor are functions of the degree of cure based on eqn (8).^36^ For a given value *α*, a plot of ln *β versus* 1/*T*_*α*_ gives the straight line, and the activation energy can be calculated from the slope.8
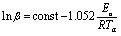


## 3. Experimentation

### 3.1. Materials

**Fig. 1:**
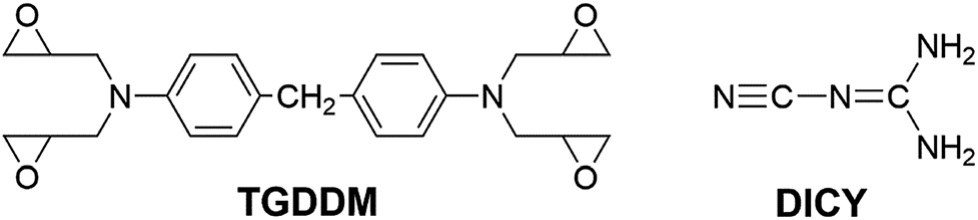
The chemical structures of TGDDM and DICY.

The epoxy resins employed in this study are *N*,*N*,*N*′,*N*′-tetraglycidyl-4,4′-diaminodiphenylmethane (TGDDM) with epoxy value of 0.75 mol/100 g and diglycidyl ether of bisphenol-A (DGEBA) with epoxy value of 0.51 mol/100 g purchased from Zhejiang Golden Roc Chemical Co., Ltd. The latent curing agent used is dicyanodiamide (DICY) supplied by Shanghai EMST Electronic Material Co., Ltd., its particle size is lower than 5 μm and the purity exceed 98%. These chemicals were used as received. The chemical structures of TGDDM and DICY are shown in Fig. 1. All other reagents and solvents were provided by Sinopharm Chemical Reagent Beijing Co., Ltd and used as received.

### 3.2. Sample preparation

The TGDDM/DICY prepolymer was prepared by mixing stoichiometric TGDDM and DICY at 80 °C under vigorous mechanical stirring for about 15 min, then a light yellow viscous mixture was obtained. The mixture was transferred into a vacuum oven at 80 °C for 15 min to drive away the entrapped bubbles. Finally, the prepolymer was prepared and characterized.

### 3.3. Characterization

The Fourier transform infrared (FTIR) spectra of TGDDM/DICY system in different curing stages were recorded on a Thermal Electron Avatar 380 infrared spectrometer. Potassium bromide (KBr) slices were prepared at a sample/KBr ratio of 100 : 1. The spectra were collected with a resolution of 4 cm^−1^ in the range of 400–4000 cm^−1^ at room temperature.

All the differential scanning calorimetry (DSC) experiments were performed on a Netzsch DZG-204F1 instrument. At first, the instrument was calibrated with purity indium standard at different heating rates. Then, a small quantity of sample (about 5 mg) was enclosed into a sealed aluminum pan and used for the DSC analysis. The specimens were heated from 30 °C up to 400 °C with different heating rates (5, 10, 15 and 20 °C min^−1^) in a nitrogen atmosphere at a constant flow of 60 ml min^−1^. Runs were carried out using an identical empty crucible as reference.

Viscosity was determined on a Brookfield CAP 2000^+^ cone-and-plate viscometer. Rotor 3 was used and its rotate speed was 750 rpm. The specimens were heated from 60 °C up to 130 °C with the heating rate of 5 °C/30 s and a series of data points were obtained every 5 °C. The tensile and shear property of the samples was measured by a Zhongzhi CZ-8000 universal testing apparatus at a crosshead speed of 10 mm min^−1^. The samples were smeared on one side of the sheet steels (10 cm × 2.5 cm × 0.2 cm) and two sheet steels were lapped together with overlapping area of 2.5 cm × 1.25 cm. Each specimen needed to experience heat preservation of 15 min under the test temperature before test and average of three individual determinations was used. Dielectric property of the samples was studied on a Tonghui Electronics TH2828S Automatic Component Analyzer at room temperature in the frequency range of 10–1000 kHz, the size of each sample was approximately 15 mm × 15 mm × 2 mm. The dielectric constant *ε* was then calculated using the equation:*ε* = *kC*_p_*d*/*ε*_0_*S*where *ε*_0_ is the vacuum dielectric constant = 8.85 × 10^−12^ F m^−1^, *C*_p_ is the capacitance value of the sample, *S* is the surface area of the sample = 2.25 × 10^−4^ m^2^, *d* is the sample thickness, and *k* is the correction factor of the instrument = 3.15. The capacitance value was directly obtained from the instrument. The water absorption was determined by weighing the change of specimens before and after being immersed in distilled water at room temperature for 24 h and average of five individual determinations was used.

## 4. Results and discussion

### 4.1. FTIR characterization of cure reaction

**Fig. 2:**
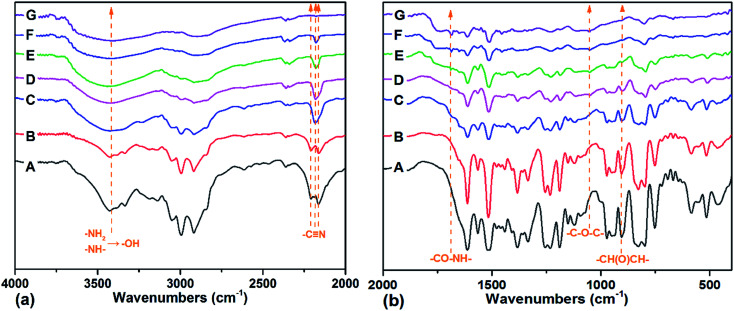
FTIR spectra of TGDDM/DICY system in different curing stages. ((a)4000–2000 cm^−1^; (b) 2000–400 cm^−1^) (A) prepolymer, (B) 100 °C/1 h, (C) 100 °C/1 h + 130 °C/1 h, (D) 100 °C/1 h + 130 °C/1 h + 160 °C/1 h, (E) 100 °C/1 h + 130 °C/1 h + 160 °C/1 h + 190 °C/1 h, (F) 100 °C/1 h + 130 °C/1 h + 160 °C/1 h + 190 °C/1 h + 220 °C/1 h, (G) 100 °C/1 h + 130 °C/1 h + 160 °C/1 h + 190 °C/1 h + 220 °C/1 h + 250 °C/1 h, respectively.

In this study, TGDDM/DICY prepolymer underwent the cure process in an air convection oven with the following temperature programs: 100 °C/1 h + 130 °C/1 h + 160 °C/1 h + 190 °C/1 h + 220 °C/1 h + 250 °C/1 h. FTIR spectroscopy was employed to monitor the dynamic changes of the characteristic absorption bands of various reactions involved in TGDDM/DICY polymerization process under the above-mentioned different curing stages. Fig. 2 summarized the FTIR spectra of TGDDM/DICY system under a sequential cure condition. The spectra were shifted along the ordinate for clarity.

**Fig. 3:**
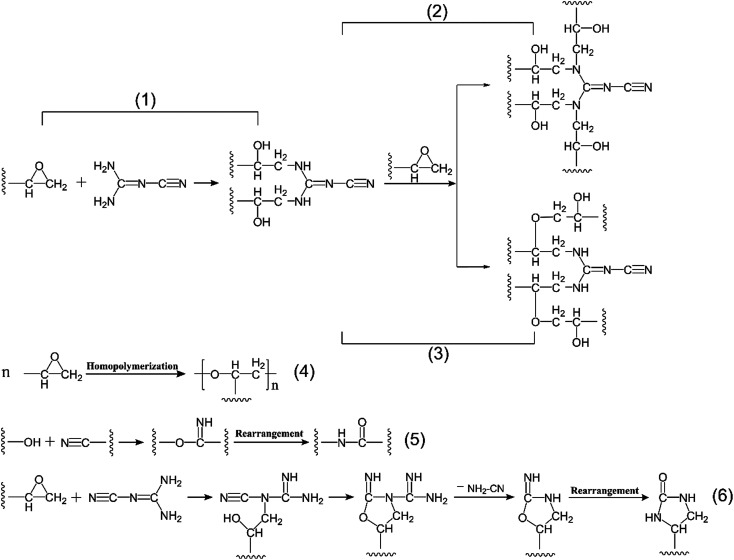
Proposed curing reactions of TGDDM/DICY system.

In the reaction stages from A to D, with the increasing temperature and polymerization time, the absorption band at 906 cm^−1^, attributable to the epoxy group (–CH(O)CH–) in epoxy resin TGDDM, decreased constantly. In the meantime, the sharp peaks around 3426 cm^−1^ and 3385 cm^−1^ ascribed to the primary amine and secondary amine in DICY developed into a broad peak around 3450 cm^−1^ associated with hydroxyl group. According to the above transformations, it was reasonable to deduce that the ring-opening addition reaction between epoxy group in TGDDM and active hydrogen of primary amine and secondary amine in DICY to produce the hydroxyl group happened in these stages. Moreover, it was noteworthy that the nitrile group (–C≡N) presented two characteristic peaks at 2206 cm^−1^ and 2162 cm^−1^ at the initial reaction stages of (A) and (B), but then it exhibited only one absorption peak at 2184 cm^−1^ during the subsequent curing process. This phenomenon was caused by the change of molecular structure environment around nitrile group resulted in a decline in the number of characteristic peaks of nitrile group from two to one. After consulting Sadtler standard infrared spectrograms,^37^ we found that if there were both nitrile group and carbon–nitrogen double bond (–C=N) in the molecular structure, only when the carbon–nitrogen double bond directly connected with the primary amine, the infrared absorption peak of nitrile group split up into two peaks around 2200 cm^−1^ owing to the Fermi resonances. However, if the carbon–nitrogen double bond directly linked up with the secondary amine, nitrile group only showed one infrared characteristic peak at about 2190 cm^−1^ as Gu X. *et al.*^24^ have demonstrated. Similarly, according to the change of infrared peak number of nitrile group, it was well-founded to speculate that the ring-opening reaction of primary amine in DICY with epoxy group in TGDDM was predominant in the lower temperature stages of A and B (Fig. 3(1)), after that further ring-opening reaction between secondary amine and epoxy group proceeded at C and D stages (Fig. 3(2)). On the other hand, a weak peak around 1052 cm^−1^ ascribed to the ether bond (–C–O–C–) appeared in the stage D, due to the etherification of epoxy group with the resultant hydroxyl group (Fig. 3(3)), and this reaction was subordinate judging from the feeble intensity of the characteristic peak. Therefore, summarizing the above analyses, the cure reaction at the reaction stages from A to D progressed mainly *via* ring opening polyaddition reaction between active hydrogen of primary amine and secondary amine in DICY and epoxy group in TGDDM.

As curing reaction went on from E to G, the absorption peak of hydroxyl group underwent a stepwise reduction in intensity, the single peak around 2184 cm^−1^ associated with nitrile group also decreased progressively and disappeared finally, and the characteristic peak of carbonyl group in amide bond emerged at 1687 cm^−1^ and enhanced gradually until the end of the reaction. These variations implied that both hydroxyl group and nitrile group participated in the subsequent reaction and form amide structure in the end. It could be conjectured by the following two successive reactions: the addition reaction of nitrile group in DICY and hydroxyl group formed in previous reactions to generate imine structure and then the rearrangement reaction of imine structure to yield amide structure (Fig. 3(5)). In this experiment, TGDDM reacted with stoichiometric DICY, no superfluous epoxy groups could homopolymerize at higher temperature in theory. Besides, the absorption peak of epoxy group disappeared at the stage of E and the intensity of ether bond peak had no obvious change in the later stages from E to G, thus the homopolymerization of unreacted epoxy group (Fig. 3(4)) can be negligible. If the reaction between nitrile group of DICY and unreacted epoxy group occurred as Fig. 3(6) shown, the characteristic peak of nitrile group was supposed to exist in the end of the reaction, since nitrile group was produced in the process. However, the opposite was true, so the reaction between nitrile group and epoxy group was inexistent in this study. Taking into consideration the above information, the reaction between nitrile group and hydroxyl group to create amide performed primarily in the reaction stages from E to G.

In a word, there were two main reactions actualized in the process of epoxy resin TGDDM cured with stoichiometric DICY. The epoxy–amine ring-opening polyaddition reaction happened first between active hydrogen in DICY and epoxy group in TGDDM. Then, the reaction between the nitrile group of DICY and the generated hydroxyl group to form the amide structure proceeded sequentially.^38^

### 4.2. DSC characterization of cure reaction

**Fig. 4:**
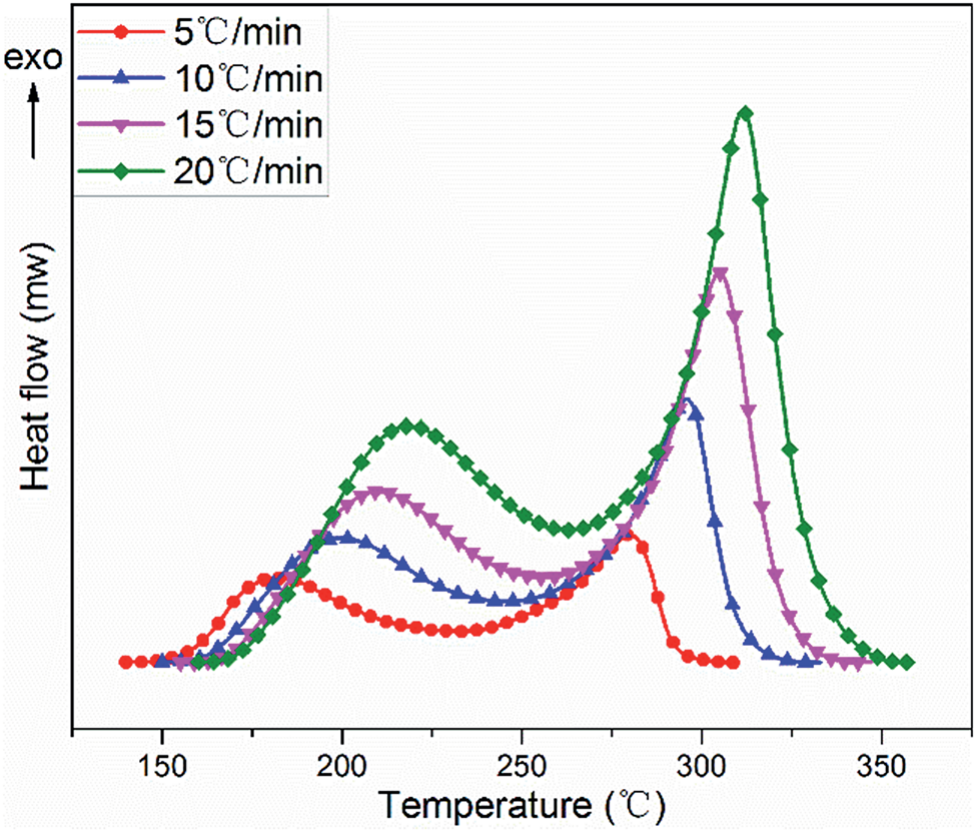
DSC thermographs for TGDDM/DICY system at different heating rates.

The non-isothermal curing reaction of TGDDM cured with stoichiometric DICY was investigated by DSC at the heating rates of 5, 10, 15 and 20 °C min^−1^. The obtained DSC thermographs of heat flow as a function of temperature at different heating rates were presented in Fig. 4 and the corresponding analytical results were listed in Table 1. Each DSC curve exhibited two overlapped exothermic peaks irrespective of the heating rates, which suggested that TGDDM/DICY system experienced a two-stage exothermic reaction and no further curing reaction occurred in the higher temperature region from a macroscopic perspective. Moreover, heating rate had a great influence on the shape of exothermic curve. As the heating rate increased, all exothermic peaks shifted to higher temperature range with expanded peak width, and the initial curing temperature, peak temperature, final curing temperature and the cure temperature range of the studied system enhanced and the cure duration decreased. These were because the heat flow increased with the increasing heating rate, the thermal effect in the unit time strengthened and the temperature difference enlarged as well. Therefore, the exothermic peak of the curing reaction moved to high temperature zone and the curing reaction was accelerated.

**Table 1:** The DSC analyses of TGDDM/DICY system at different heating rates

*β* (°C min^−1^)	*T*_i_ a (°C)	*T*_p1_ b (°C)	*T*_p2_ c (°C)	*T*_f_ d (°C)	Cure duration^e^ (min)	Cure range^f^ (°C)	Δ*H*_1_^g^ (J g^−1^)	Δ*H*_2_^h^ (J g^−1^)	Δ*H*^i^ (J g^−1^)
5	140	185	277	310	33.6	170	446	421	867
10	150	201	296	333	17.9	183	429	370	799
15	155	211	305	347	12.5	192	423	374	797
20	160	220	313	358	9.6	198	484	375	859

a The initial curing temperature.

b The first exothermic peak temperature.

c The second exothermic peak temperature.

d The final curing temperature.

e Total elapsed time of cure reaction.

f Total temperature ranges of cure reaction, equal to the difference between *T*_i_ and *T*_f_.

g The reaction enthalpy of the first exothermic peak.

h The reaction enthalpy of the second exothermic peak, respectively.

i The total enthalpy of cure reaction.

Integration of the two exothermic peaks with respect to the linear baseline gave rise to the values of total reaction enthalpy Δ*H* for different heating runs. The total curing reaction enthalpy, which ranged from 797 to 867 J g^−1^ within the experimental errors limit (5% of the average value), was independent of the heating rate. This observation implied that when the heating rate ascended TGDDM/DICY system could still be cured at a faster speed, encountered the identical main chemical reactions and finally reached essentially the same reaction extent. These findings are similar to what have been reported in some other studies.^39,40^

The ranges of peak temperature and reaction enthalpy of the first and second exothermic peaks at different heating rates were 185–220 °C, 423–484 J g^−1^ and 277–313 °C, 370–421 J g^−1^, respectively. The reaction enthalpy (423–484 J g^−1^) of the first exothermic peak was within the typical value range for many epoxy–amine systems, which was practically acceptable compared with other polyamine epoxy curing agents.^41,42^ This finding suggested that the first exothermic peak was attributed to the reaction between the epoxy group of TGDDM and the amino group of DICY, which is consistent with the aforementioned results proposed by FTIR analyses.

**Fig. 5:**
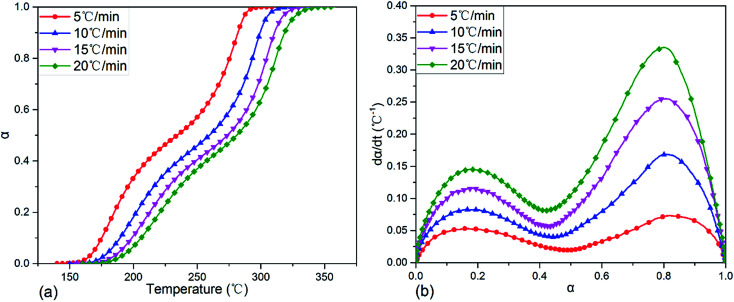
Plots of fractional conversion *vs.* temperature (a) and reaction rate d*α*/d*t vs.* fractional conversion (b) for TGDDM/DICY system at different heating rates.

After transforming the original DSC data on exothermic curves in Fig. 4 with eqn (1) and (2), the variations of fractional conversion *α* with temperature and reaction rate d*α*/d*t* with *α* at various heating rates were obtained and the corresponding plots were indicated in Fig. 5(a) and (b), respectively. It was clear in Fig. 5(a) that each fractional conversion grew very slowly at the beginning of curing reaction. After heated to the given temperature, the fractional conversion performed a sharp increase and its increment speed was not a constant which enhanced fast at first, then slowed down, after that it became much faster again. In the end, the fractional conversion leveled off at a certain value. Moreover, increasing the heating rate caused the fractional conversional curve shifting to a higher temperature range and becoming steeper. This indicated that to acquire the same fractional conversion and achieve identical reaction extent of TGDDM/DICY system, the non-isothermal curing reaction temperature needed to be raised with the increasing heating rate.

The relationship between reaction rate d*α*/d*t* and fractional conversion *α* of TGDDM/DICY system was depicted in Fig. 5(b). The curing reaction rate improved with the increase of heating rate throughout the entire fractional conversion. As the fractional conversion enhanced, each reaction rate curve run out from peak to trough, then to peak again and finally inclined to zero when the curing reaction completed. All reaction rate curves exhibited two peaks and the two peak values of d*α*/d*t* were at some intermediate values rather than the initial values of *α*. That is, the fastest reaction rates of the two exothermic reactions existed in some intermediate process of the curing reaction instead of the initial stage, which implied that TGDDM/DICY system experienced two autocatalytic curing processes. The first and second autocatalytic curing processes were corresponding to the first and second curing exothermic peaks of the DSC curves in Fig. 4, respectively. Therefore, the research on curing kinetics of TGDDM/DICY system ought to be divided into two stages: the first stage when the fractional conversion was less than 0.45 and the second stage when the fractional conversion was greater than 0.45. The first and second peak values of d*α*/d*t* appeared at nearly the same fractional conversions (*α* = 0.16–0.18 and 0.80–0.82) irrespective of the different heating rates, which suggested that the heating rate had a large impact on the curing kinetics, but hardly affected the basic reaction mechanism in the first and second non-isothermal curing processes. Furthermore, the model-free kinetic method was suitable for the whole experiment and the activation energies at the first and second peak values of d*α*/d*t* should be identical despite of the diverse heating rates.^43^

### 4.3. Activation energy of the non-isothermal cure

**Fig. 6:**
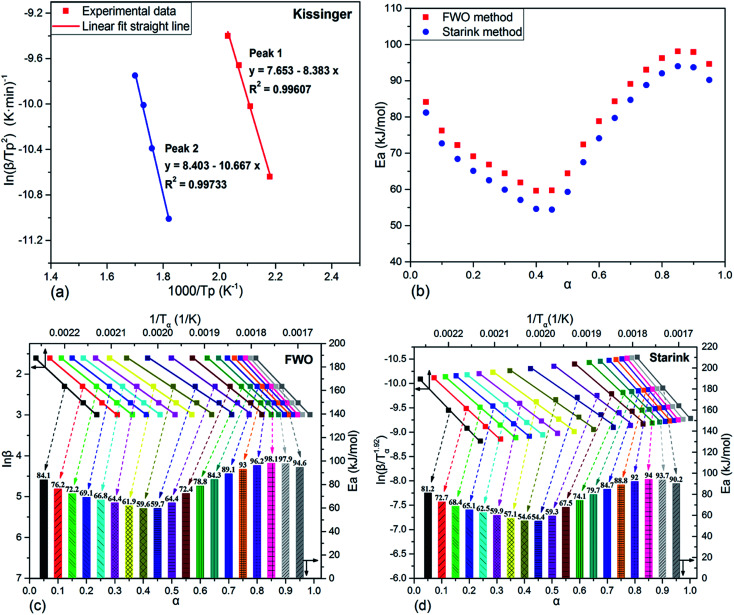
Activation energy analyses of TGDDM/DICY system under different methods ((a) Kissinger plots of the two cure processes; (b) variation of activation energy *versus* fractional conversion from FWO method and Starink method; (c) FWO plots at different fractional conversions; (d) Starink plots at different fractional conversions).

The kinetic analyses of the above two curing reaction stages were conducted by Kissinger method based on the multiple heating rates firstly. The typical Kissinger plots of the two cure processes were drawn in Fig. 6(a) and the calculated activation energy values were 69.7 and 88.7 kJ mol^−1^, respectively. The *E*_a_ value 69.7 kJ mol^−1^ of the first curing reaction stage was in the typical range of epoxy–amine polymerizations, likely indicating that the essential ring-open mechanism of the epoxy–amine reaction took place in the first curing process.^41,42,44^

Although the Kissinger method has been applied for kinetic studies, it only produces two single *E*_a_ values from the two maximum reaction rates of the two DSC exothermic peaks for the whole reaction process. The obtained results are believable only when *E*_a_ values are two unequal constants throughout the two different reaction processes.^45–47^ As aforementioned, the curing of epoxy resin consists of many elementary reactions, complex mass transfer and phase transition processes which leads to a complicated curing reaction mechanism. Therefore, the activation energy changes with fractional conversion during the curing reaction and no variation of *E*_a_ value with cure reaction progress is unrealistic. Fortunately, the isoconversional kinetic analysis, without assuming any specific kinetic models, can provide us with more straightforward information concerning thermosetting curing reactions, more particularly, which can reveal a dependence of activation energy on fractional conversion. Analysis of this dependence contributes to untangling complex mechanism of cure processes and predicting kinetics. Herein, integral model free isoconversional methods, Flynn–Wall–Ozawa (FWO) and Starink (the modified Kissinge–Akahira–Sunose) methods, are frequently used in present computations of the activation energy at different fractional conversion.

Fig. 6(b)–(d) displayed the activation energy variation analyzed by FWO and Starink methods and the corresponding results which were divided into two parts according to the two curing reaction stages were also listed in Table 2. Clearly, activation energy calculated from FWO and Starink methods varied greatly with fractional conversion, which exposed that the non-isothermal curing reaction of TGDDM/DICY seemed to follow the multi-step reaction mechanisms associated with different kinetic steps with varying energetic barriers. The detailed discussion on this correlation was as follows. As seen in Fig. 6(b), at the first curing reaction stage (*α* < 0.45) the activation energy investigated by FWO method decreased from 84.1 kJ mol^−1^ to 59.6 kJ mol^−1^, which could be elucidated as follows. First, the –OH functionalities generated from epoxy–amine ring-opening reaction can markedly catalyze the remaining epoxy–amine reaction *via* a trimolecular transition state, particularly an activated epoxy–amine–hydroxyl complex, whereby the energetic barrier for the epoxy–amine reaction lowered.^48–50^ Second, the molecular weight increased slowly at low fractional conversion, but the viscosity of reaction mixture dropped dramatically at this stage due to the rising temperature, the elevated mobility of polymer chain segments and the growing effective collision of molecular reaction groups further reduced the energetic barrier for the diffusion of reactive species.^51^ Consequently, superposition of the above effects led to the decline of the overall activation energy at the first curing reaction stage.

**Table 2:** The values of activation energy obtained by FWO and Starink method at different fractional conversions

The first curing reaction stage					The second curing reaction stage				
*α*	*E*_a-FWO_, kJ mol^−1^	*R*_FWO_^2^ a	*E*_a-Starink_, kJ mol^−1^	*R*_Starink_^2^	*α*	*E*_a-FWO_, kJ mol^−1^	*R*_FWO_^2^	*E*_a-Starink_, kJ mol^−1^	*R*_Starink_^2^
0.05	84.1	0.99888	81.2	0.99862	0.50	64.4	0.99391	59.3	0.99233
0.10	76.2	0.99936	72.7	0.99916	0.55	72.4	0.99660	67.5	0.99584
0.15	72.2	0.99982	68.4	0.99976	0.60	78.8	0.99744	74.1	0.99692
0.20	69.1	0.99990	65.1	0.99986	0.65	84.3	0.99832	79.7	0.99802
0.25	66.8	0.99978	62.5	0.99974	0.70	89.1	0.99896	84.7	0.99880
0.30	64.4	0.99958	59.9	0.99954	0.75	93.0	0.99950	88.8	0.99944
0.35	61.9	0.99860	57.1	0.99834	0.80	96.2	0.99982	92.0	0.99980
0.40	59.6	0.99696	54.6	0.99624	0.85	98.1	0.99980	94.0	0.99976
0.45	59.7	0.99367	54.4	0.99194	0.90	97.9	0.99990	93.7	0.99988
Mean	68.2		64.0		0.95	94.6	0.99938	90.2	0.99920
					Mean	86.9		82.4	

a *R* ^2^, coefficient of determination.

During the second curing reaction stage (*α* > 0.45), as *α* continued increasing, the activation energy firstly exhibited a sharply growth and reached a maximum value 98.1 kJ mol^−1^ at *α* = 0.85. This observation may indicated that the reaction still performed in the reaction-controlled regime and determined the overall reaction kinetics. As the subsequent reactions went on and the curing degree progressively deepened, the propagating molecular chain continuously increased the molecular weight, which notably enhanced the viscosity of the reaction mixture. Then, the free volume only allowed local motions of the chain segments. Therefore, a great degree of cooperativity among the chain segments was required to initiate translational motion of the segments,^52^ which gave rise to a large energy barrier of the segment motion and increased the overall activation energy. Finally, as the reaction progressed in the deep-conversion range (*α* > 0.85), the activation energy decreased again. This fact implied the rate-determining step of the reaction generally changed from the reaction control to the diffusion limitation. The reason lied in that the mobility of the molecular chains carrying the reactive species became more and more limited due to the increased junction points and the gradually elevated glass temperature, which greatly restricted configuration rearrangements and cooperative motions of the network chains, especially as the reaction system approached its glassy state.^53^

As illustrated in Fig. 6(b) and Table 2, the activation energies obtained from FWO and Starink methods presented almost the same changing tendency and possessed approximate values at each conversion. The mean value of activation energies estimated by FWO method in the first and second curing stages was 68.2 and 86.9 kJ mol^−1^, which were comparable to the results (69.7 and 88.7 kJ mol^−1^) acquired *via* Kissinger method. Therefore, the Kissinger method, which considered the specific activation energy obtained from the maximum reaction rate as the average activation energy for the whole reaction process, was appropriate for the curing kinetics study of TGDDM/DICY system.

## 5. Properties of TGDDM/DICY system

The viscosity, tensile and shear property, dielectric property and water absorption of the TGDDM/DICY system were studied in this work, for comparison, the DGEBA/DICY system was prepared by stoichiometric DGEBA and DICY under the same reaction conditions.

**Fig. 7:**
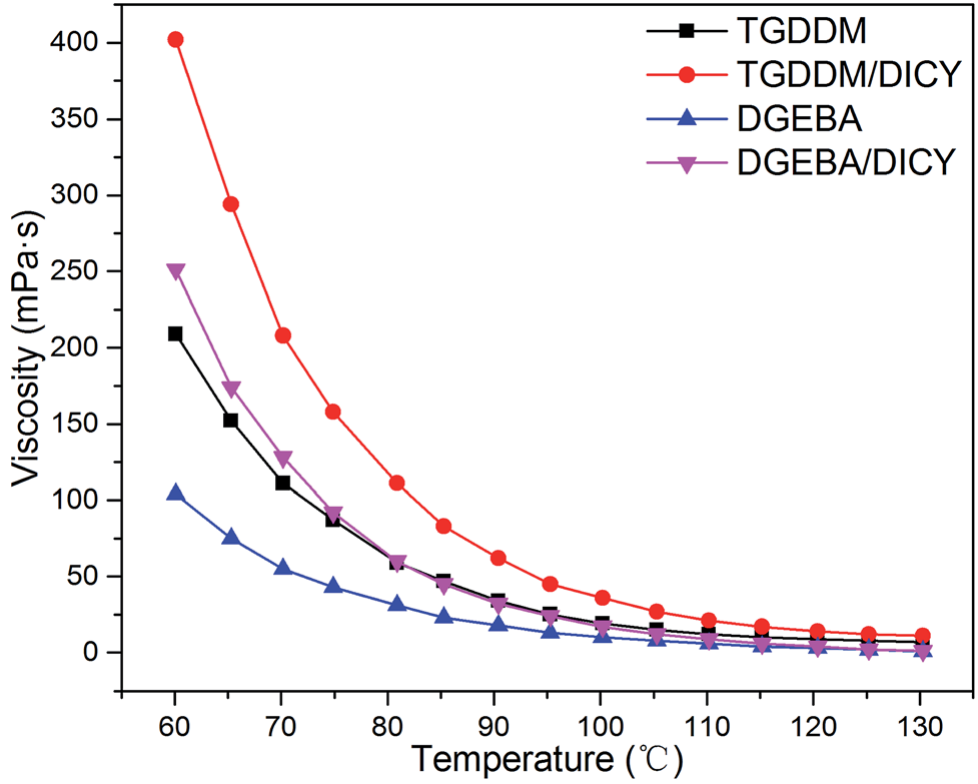
Viscosity curves of TGDDM/DICY and DGEBA/DICY systems.

The dependences between viscosity and temperature of pure TGDDM, TGDDM/DICY prepolymer, pure DGEBA and DGEBA/DICY prepolymer were given in Fig. 7. The viscosities of all specimens decreased with the increasing temperature, resulting from the more fierce molecular thermal motion after heating up, which led to a decline in viscosity on macro level. Owing to the nonlinear multifunctional glycidyl groups of TGDDM, TGDDM/DICY with larger molecular weight possessed higher viscosity than DGEBA/DICY. As temperature increased, the variation tendency of viscosities of both TGDDM/DICY and DGEBA/DICY did not encountered a turning point, did not decrease first and then increase. This suggested that the cure reaction speed of the two systems cured with DICY were slow in the range of test temperature, the effect of the enhancement of molecular weight as reaction proceeded which brought about the increase of viscosity did not play a dominant role. It is well proved that the latent curing agent DICY can endow its epoxy prepolymers with excellent room-temperature stability and long shelf life.

The tensile and shear strength, dielectric constant and water absorption of the cured TGDDM/DICY and DGEBA/DICY were listed in Table 3. As anticipated, the cured TGDDM/DICY owned favorable tensile and shear strength of 12.6–27.1 MPa at 25–200 °C, which had a distinct advantage over the cured DGEBA/DICY. It was the higher crosslink density of TGDDM/DICY because of the nonlinear multifunctional groups and the weaker rigid chain skeleton of DGEBA/DICY resulting from the ether linkage in DGEBA that contributed to a better tensile and shear property of TGDDM/DICY than DGEBA/DICY. At 200 °C, the tensile and shear strength of TGDDM/DICY could still reach 12.6 MPa, which showed that the cured TGDDM/DICY own outstanding heat resistance and adhesive property, especially to steel surface.

**Table 3:** Tensile and shear property, dielectric property and water absorption of TGDDM/DICY and DGEBA/DICY systems

Properties		TGDDM/DICY	DGEBA/DICY
*T*_S_ a (MPa)	25 °C	27.1	19.6
	100 °C	25.6	17.4
	150 °C	18.4	13.7
	180 °C	15.1	9.6
	200 °C	12.6	7.1
*ε* b	10 kHz	3.28	3.22
	100 kHz	3.27	3.19
	1000 kHz	3.26	3.17
*w* c (%)		0.41	0.47

a *T*_S_: tensile and shear strength.

b *ε*: dielectric constant.

c *w*: water absorption.

The dielectric constants of the cured TGDDM/DICY and DGEBA/DICY were 3.26 and 3.17 at 1000 kHz, respectively, and DGEBA/DICY showed lower dielectric constant than TGDDM/DICY in the frequency range of 10–1000 kHz. This result was caused by the existence of the pendant CH_3_ groups in DGEBA, which prevent the chain packing and increased the free volume. For both systems, with increasing the frequency from 10 to 1000 kHz, the dielectric constant decreased. These variations were attributed to the frequency dependence of the polarization mechanisms.

The water absorption values of the cured TGDDM/DICY and DGEBA/DICY were 0.41% and 0.47%, respectively, and TGDDM/DICY presented lower water absorption than DGEBA/DICY. It might be attributed to the high crosslink density of the cured TGDDM/DICY system, which possessed low porosity and inhibited the absorption of moisture molecules on the polymer surfaces.

## 6. Conclusions

In this work, a novel epoxy resin was developed by multifunctional epoxy resin *N*,*N*,*N*′,*N*′-tetraglycidyl-4,4′-diaminodiphenylmethane (TGDDM) and stoichiometric latent curing agent dicyandiamide (DICY). The curing reaction mechanism and non-isothermal cure behaviors of the mixture were monitored by FTIR and DSC techniques, respectively. According to the FTIR observations, the curing mechanism of TGDDM/DICY system consisted of two main reactions as follows. The ring-opening reaction between active hydrogen of amino group in DICY and epoxy group in TGDDM and the reaction between nitrile group of DICY and the generated hydroxyl group to yield the amide structure. Based on the analyses of the DSC thermogram, the blends exhibited two different cure regimes in the temperature range of 140–358 °C and experienced two autocatalytic curing processes with *α* = 0.45 as the boundary, the corresponding activation energies evaluated by Kissinger method were 69.7 and 88.7 kJ mol^−1^, respectively. The total curing reaction enthalpy (797–867 J g^−1^) was independent of the heating rate. On the other hand, the dependency between activation energy and fractional conversion was determined by applying model-free isoconversional analysis with the Flynn–Wall–Ozawa (FWO) and Starink methods. Results showed that both two methods revealed similar tendencies and possessed approximate values at each fractional conversion, and activation energy varied greatly with fractional conversion. In general, the reaction control took the dominant role in the overall kinetics during the early stage of the reaction and the viscosity and autocatalysis were moderately influential, whereas in the deep-conversion region (*α* > 0.85) the diffusion limitation became much predominant. The resulting cured TGDDM/DICY presented excellent mechanical, adhesive and dielectric properties and lower water absorption.

## Conflicts of interest

There are no conflicts to declare.

## Supplementary Material

RA-008-C7RA13233F-s001
