# Supplementary material for: Reaction mechanism, cure behavior and properties of a multifunctional epoxy resin, TGDDM, with latent curing agent dicyandiamide
Source: RSC Adv. 2018 Feb 21;8(15):8248–58. doi: 10.1039/c7ra13233f (PMC9078540; doi:10.1039/c7ra13233f)
Supplement: RA-008-C7RA13233F-s001 [file RA-008-C7RA13233F-s001.pdf]

Electronic Supplementary Material (ESI) for RSC Advances.
This journal is © The Royal Society of Chemistry 2018

Mechanism of model phenyl glycidyl ether/dicyandiamide reaction

The main impetus of this investigation was to test the validity of the mechanism proposed from FTIR results for TGDDM/DICY system.

To verify the reaction mechanism, NMR measurements can be used. Due to the low solubility of the products in TGDDM/DICY system, a model compound of epoxy was used for the curing system. The monofunctional epoxy compound phenyl glycidyl ether was preferred in academia.

phenyl glycidyl ether (PEG) was supplied by J&K Scientific and used as received.

A mixture of 12.01 g (0.08 mol) phenyl glycidyl ether, 1.68 g (0.02 mol) dicyandiamide were heated on a Thermocap-controlled heating mantle under reflux condenser. The temperature was maintained at 120°C for 4 h. The reaction was monitored by thin layer chromatography (TLC). Some reaction products were separated by column chromatography (eluent: tetrahydrofuran: water = 3:1) and the solvents were removed by the rotary evaporator. The isolated products were then dried overnight in a room temperature vacuum oven prior to the nuclear magnetic resonance (NMR) spectroscopy analysis.

^{1}H NMR and ^{13}C NMR spectra were obtained on a Bruker AV 400 MHz spectrometer using in DMSO-d_6 as solvent and tetramethylsilane as internal standard. Chemical shifts (δ) were expressed in ppm.

Unfortunately, it was not attainable to obtain all products in a purity and quantity sufficient enough for the exact interpretation of the NMR spectra in our experiment. Two main products were acquired and characterized by NMR. The chemical structures were shown in Fig.1. Product 1: ^{1}H-NMR (400 MHz, DMSO-d_6, ppm): 7.27 (m, 4H), 6.94 (m, 2H), 6.90 (m, 4H), 6.21 (s, 2H), 5.31 (s, 2H), 4.43 (m, 4H), 4.29 (m, 2H) and 3.59 (m, 4H); ^{13}C-NMR (400 MHz, DMSO-d_6, ppm): 163.5, 159.7, 130.3, 121.4, 118.8, 115.8, 71.3, 70.5 and 55.7. Product 2: ^{1}H-NMR (400 MHz, DMSO-d_6, ppm): 8.20 (s, 1H), 7.23 (m, 8H), 6.92 (m, 4H), 6.87 (m, 8H), 6.19 (s, 4H), 5.29 (s, 3H), 4.42 (m, 6H), 4.31 (m, 1H), 4.29 (m, 3H), 4.01 (m, 2H), 3.72 (m, 2H) and 3.61 (m, 6H); ^{13}C-NMR (400 MHz, DMSO-d_6, ppm): 169.2, 163.5, 159.5, 130.3, 121.2, 118.8, 115.7, 71.2, 70.8, 62.9, 55.5, 54.1, 50.7 and 49.7.

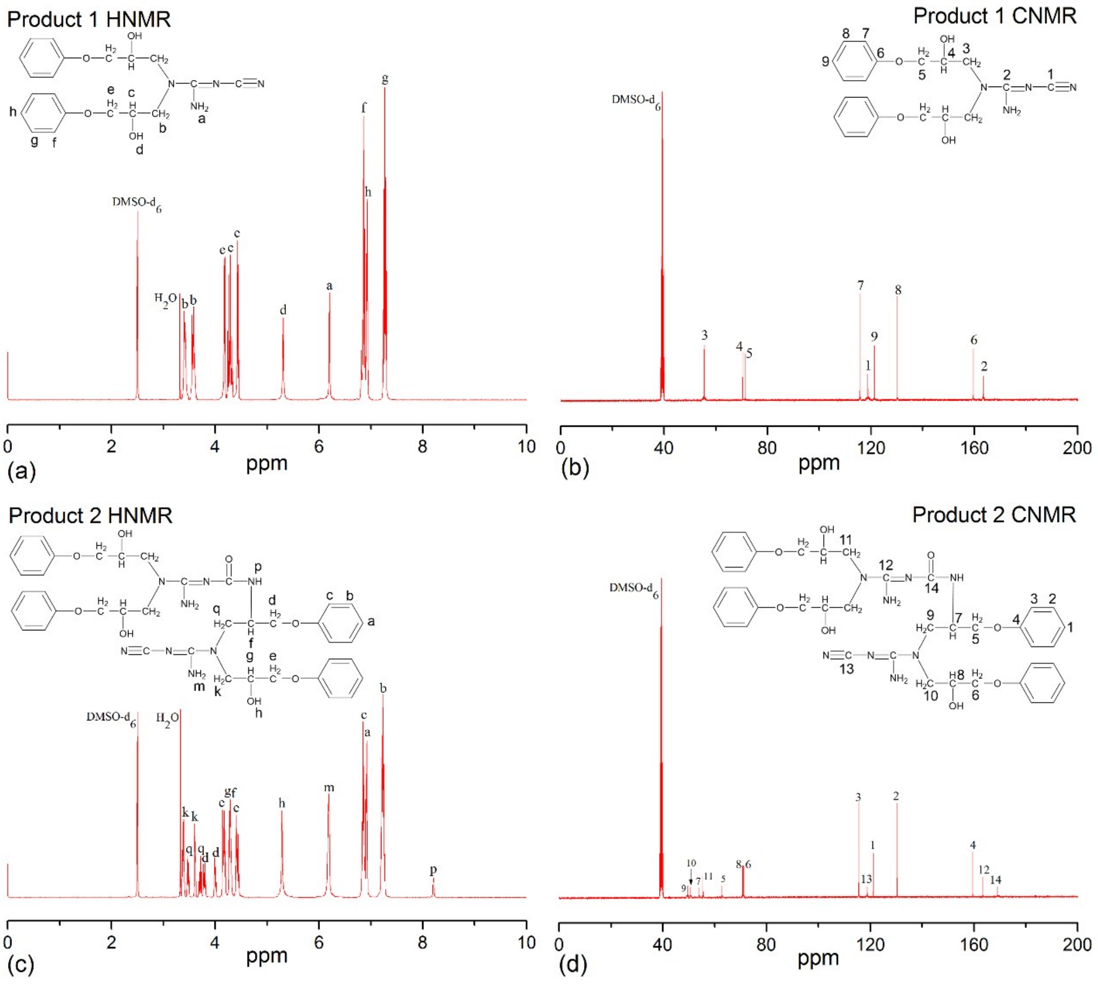

Fig. 1. NMR spectra and structures of (a) Product 1 HNMR, (b) Product 1 CNMR, (c) Product 2 HNMR, (d) Product 2 CNMR.

The chemical shifts of the four different aromatic carbon atoms in products 1 and 2 appeared at about 159.7, 130.2, 121.4 and 115.8 ppm similar to the initial PGE. The signals at 118.8 ppm for the cyano group and 163.5 ppm for the >C=N- group of products 1 and 2 were also found in the CNMR spectrum of the pure DICY. Besides, the signals at 6.57 ppm for the primary amine in DICY and at 50.4, 44.3, 3.1 and 2.6 ppm for the epoxide group in PEG encountered variations in follow-up NMR measurements. These observations implied that products 1 and 2 possessed partially similar chemical structures as reactants PEG and DICY, and the reaction occurred first between the primary amine of DICY and the epoxide group of PEG. The chemical shifts between 40 and 80 ppm were assigned to the characterization of the aliphatic carbons in products 1 and 2. Two different carbon atoms at about 55.4 and 163.5 ppm and one hydrogen proton at 3.6 in products 1 and 2 conformed to the characterization of tertiary amine group $(-CH_2)_2$>N-C⩽. The signals at 169.2 in CNMR and 8.2 ppm in HNMR of product 2 attributed to the amide group -C(O)-NH-.

Summarizing all the assignment, chemical shift and integral area of characteristic signals in HNMR and CNMR measurements, two different types of products with

tertiary amine structure and amide structure were deduced, respectively. Taking into consideration the formation of these products, a possible reaction pathway was proposed as exhibited in Fig.2. The glycidyl group in PEG first reacted with the active hydrogens of primary amine in DICY to form tertiary amine structure, than the addition reaction between nitrile group and resultant hydroxyl group proceeded to generate amide structure. It is clear that the model investigations with DICY demonstrated a similar reaction behaviour to the TGDDM/DICY system and the NMR studies were consistent with the information obtained by FTIR. The possible reaction pathway proposed from FTIR results for TGDDM/DICY system was reasonable.

It should be noted that the main reaction course of PEG/DICY is explicated in this study and a number of other intramolecular and intermolecular reactions and side reactions have not been elucidated yet. In order to describe all the complex nature of epoxy resin cured with DICY, more sophisticated analytical techniques are of great necessity and more careful investigations relating to the curing reactions need to be continued.

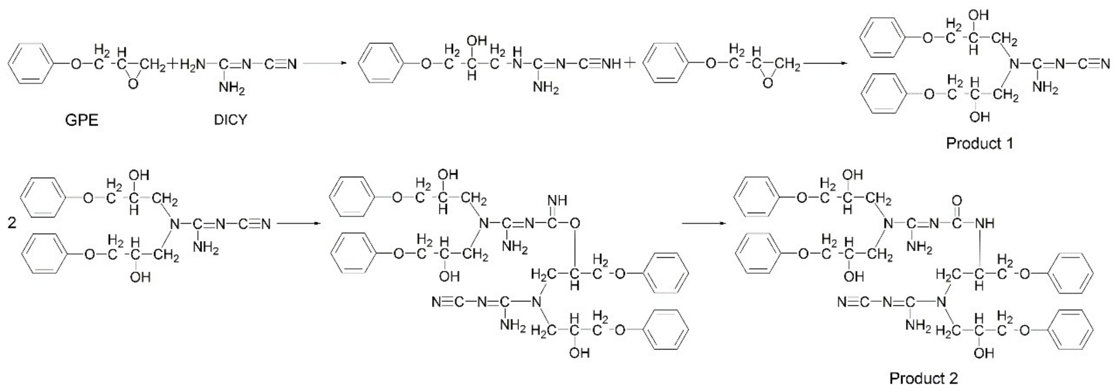

Fig. 2. Proposed reaction pathway of PEG/DICY
